# Supplementary material for: MIAT Is an Upstream Regulator of NMYC and the Disruption of the MIAT/NMYC Axis Induces Cell Death in NMYC Amplified Neuroblastoma Cell Lines
Source: Int J Mol Sci. 2021 Mar 25;22(7):3393. doi: 10.3390/ijms22073393 (PMC8038079; doi:10.3390/ijms22073393)
Supplement: Supplementary file 1 [file ijms-22-03393-s001.pdf]

Supplementary Information

Materials and Methods

Immunofluorescence Staining

For flow cytometry cells were trypsinized and fixed in 4% formaldehyde-phosphate-buffered saline for 15 min at room temperature and permeabilized by adding ice-cold 90% methanol 48 hours after transfection. Cells were then incubated with anti-NMYC rabbit monoclonal antibody diluted 1:400 in 0.5% BSA for 1 hour. Following several washing steps, cells were incubated for 30 min in the dark with Alexa Fluor 647 Goat anti-rat IgG at a dilution of 1:500. Protein expression was measured by flow cytometry - BD FACSCelesta, and data were analyzed by Flowlogic software.

Cell Transfection

The pCMV6-AC-GFP and pCMV6-AC-NMYC-GFP vectors were obtained from Origene Technologies. The vector DNAs were transfected using Lipofectamine 3000 (Thermo Fisher Scientific) according to the manufacturer's instructions. After transfection at 37 °C for 24 h, cells were selected with medium with puromycin (Thermo Fisher Scientific) for two weeks and then analyzed.

Results

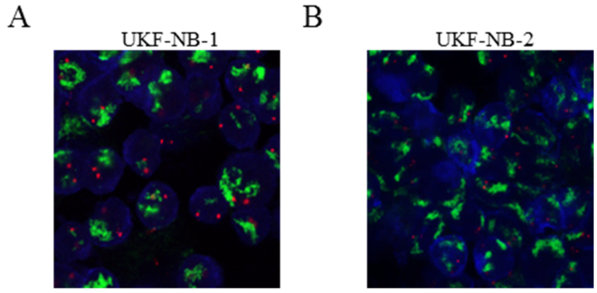

Figure S1. Representative FISH images of NBL cells. The status of *NMYC* amplification is determined by using a dual-color probe. Green signals represent the specific probe for *NMYC* and red control signals on the 2q11 probe of chromosome 2. Images show amplification of *NMYC* in (**A**) UKF-NB-1 and (**B**) UKF-NB-2 cell lines (1000x magnification).

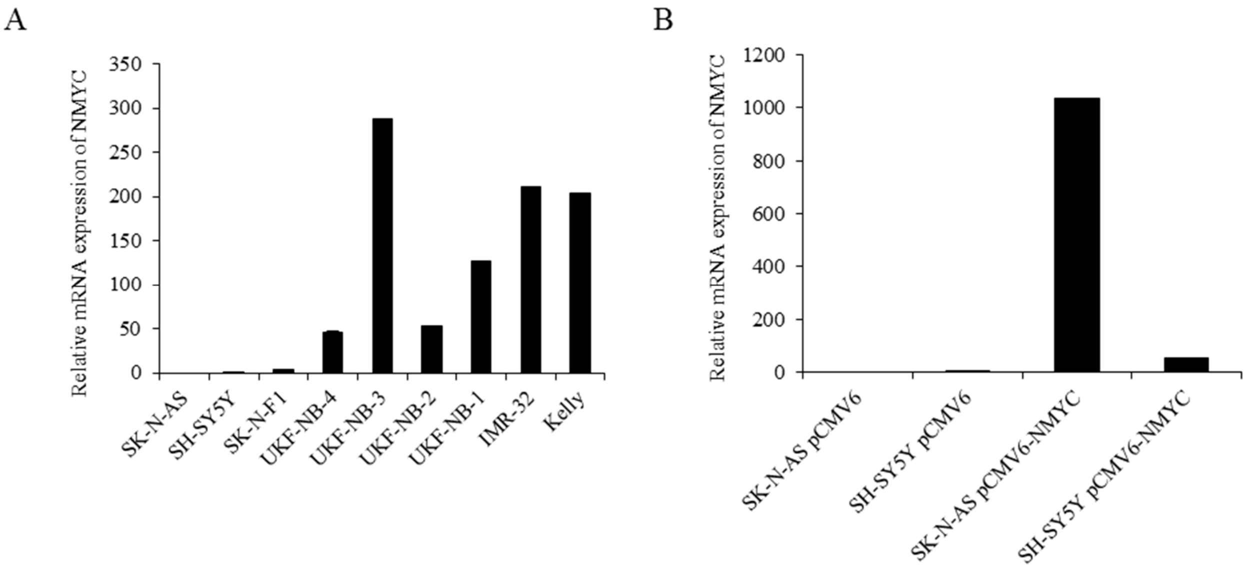

Figure S2. NMYC expression in neuroblastoma cell lines. (**A**) *NMYC* amplified cell lines have unequivocally higher expression of NMYC mRNA than non-amplified cell lines. (**B**) Similarly, high NMYC expression is in SH-SY5Y and SK-N-AS with ectopic overexpression of NMYC. The graph shows representative data of at least three independent experiments.

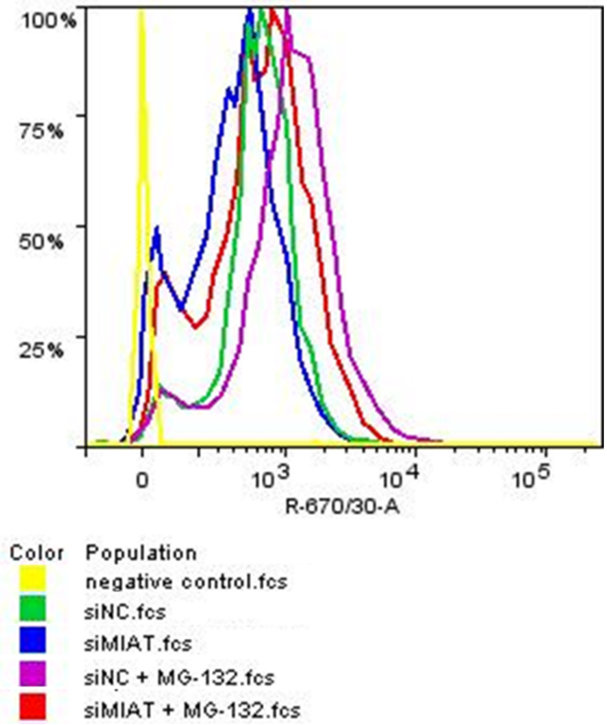

Figure S3. MIAT downregulation and simultaneous proteasome inhibition leads to decreased NMYC expression. The NMYC expression in siNC or siMIAT transfected neuroblastoma UKF-NB-4 cells and simultaneous proteasome inhibition was determined by

immunofluorescence staining and flow cytometry. The figure shows representative data of three independent experiments.

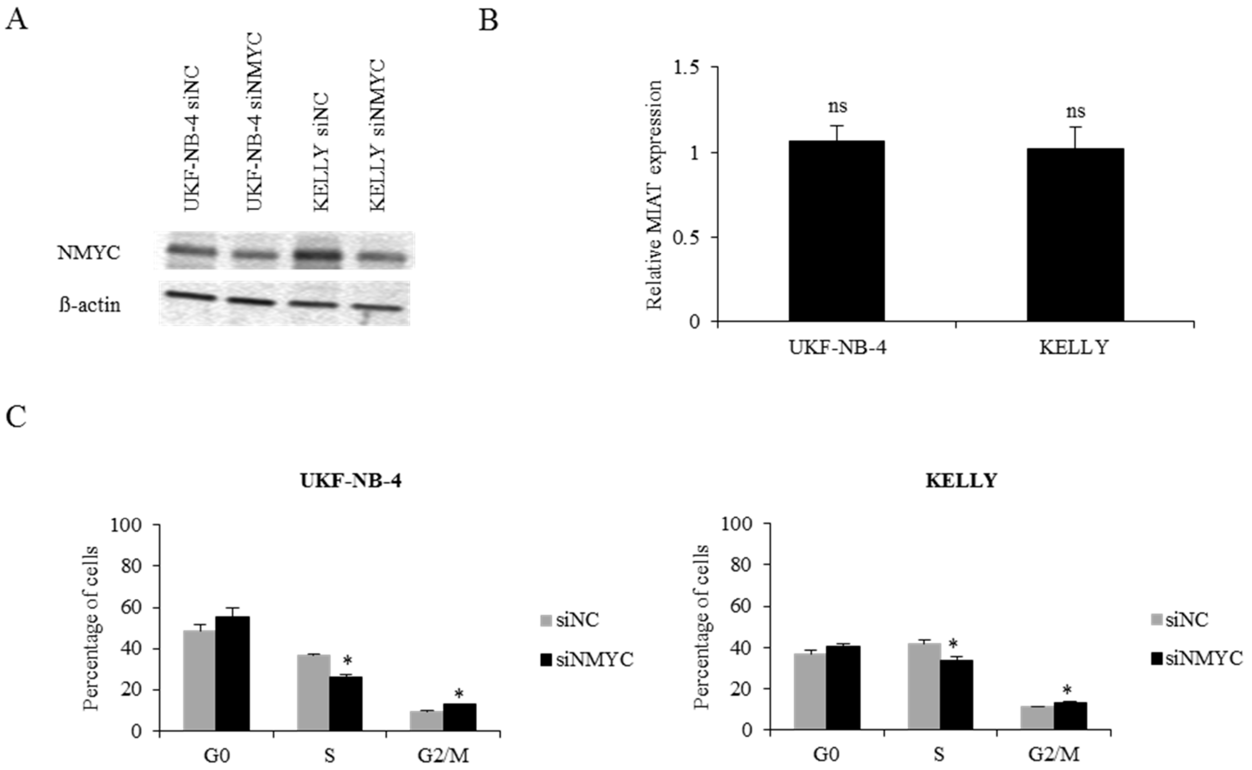

Figure S4. NMYC downregulation does not affect MIAT expression. (A) Western blot demonstrates that the NMYC expression level is decreased in UKF-NB-4 and KELLY cells transfected with siNMYC. (**B**) The MIAT expression in siNC or siNMYC transfected neuroblastoma cells was determined by qRT-PCR. (**C**) The percentage of Alexa Fluor™647 -stained cells at the G_0, S, and G_2/M phases in UKF-NB-4 and SH-SY5Y cells transfected with siMIAT and siNC were determined by flow cytometry. The figure shows representative data of three independent experiments. Values are mean ± SD from three independent experiments. ns means not significant, * $p<0.05$ as compared to siNMYC/siNC group (REST 2009; ANOVA with post-hoc Tukey HSD Test).

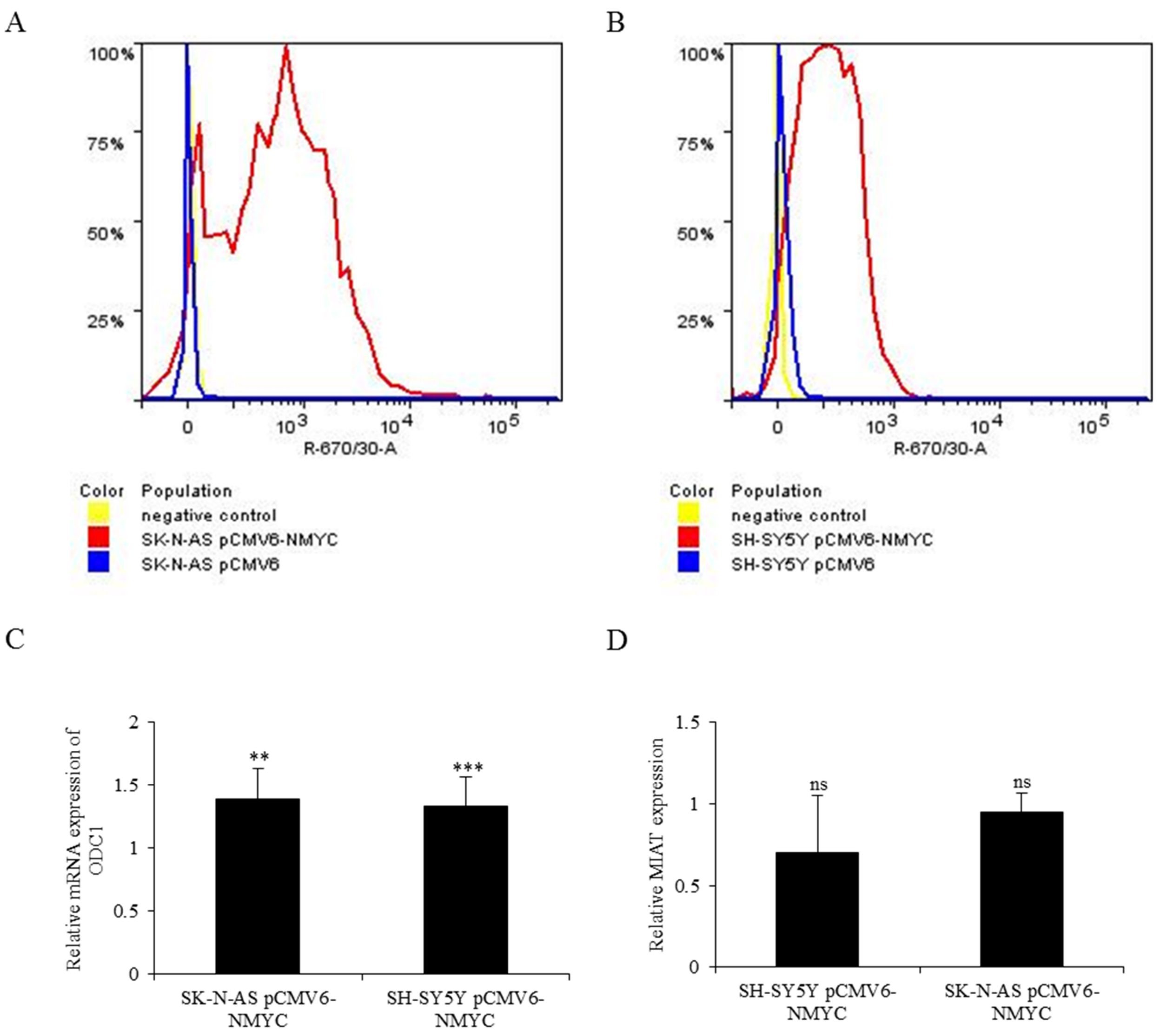

Figure S5. NMYC overexpression in NBL cell lines without *NMYC* amplification does not affect MIAT expression. The NMYC expression in NMYC or vector-transfected neuroblastoma (**A**) SH-SY5Y and (**B**) SK-N-AS cells was determined by immunofluorescence staining. (**C**) The ODC1 and (**D**) MIAT expression in NMYC or vector-transfected neuroblastoma cells were determined by qRT-PCR. The figure shows representative data of three independent experiments. Values are mean ± SD from more than three independent experiments. ns means not significant, ** $p<0.01$, *** $p<0.001$ as compared to vector/NMYC group (REST 2009; ANOVA with post-hoc Tukey HSD Test).

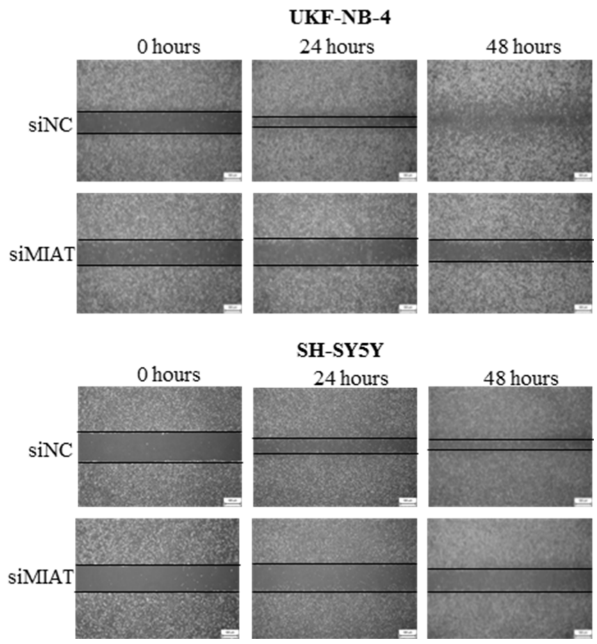

Figure S6. MIAT downregulation decreases UKF-NB-4 and SH-SY5Y cell migration. The scratch-wound gap of UKF-NB-4 and SH-SY5Y cells with or without MIAT downregulation was photographed before and after 24 and 48 hours incubation (200x magnification).

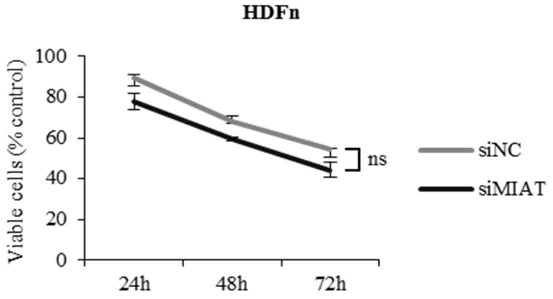

Figure S7. Viable HDFn cells after MIAT knockdown were measured by PrestoBlue at different time points. Values are mean ± SD from three independent experiments. ns means not significant as compered siMIAT/siNC group (ANOVA with post-hoc Tukey HSD Test).

Table S1. The primer sequences for qRT-PCR.

**Gene**		**Primer sequence (5′-3′)**
MIAT	forward	AAAAACAGACACGTTCATGTGGC
	reverse	AAAAACCAGGAACTTGCTGCTC
	probe	CCCCGTCGGCATCACAGG
MYC	forward	CTGGTGCTCCATGAGGAGA
	reverse	CTCTTTTCCACAGAAACAACATCG
	probe	CCGCCCACCACCAGCAG
NMYC	forward	GGACACCCTGAGCGATTC
	reverse	CTGGACTGAGCCCTCCC
	probe	CCCAAGAACGCAGCCCTGGG
ODC	forward	AGGTTGGTTTCAGCATGTATCTG
	reverse	AACGCTGGGTTGATTACGC
	probe	CCAGGAAAGCCACCGCCA
